# Diagnosis and management of COVID toes in outpatients: a case report

**DOI:** 10.1186/s13256-024-04626-9

**Published:** 2024-06-28

**Authors:** Marie Pouxe, Aziz Abdulkarim, Serge de Vallière, Teofila Seremet, Bernard Favrat, Ioannis Kokkinakis

**Affiliations:** 1 University Center for Primary Care and Public Health, Unisanté, Lausanne, Switzerland; 2 Service of Infectious Diseases, Lausanne University Hospital (CHUV), Lausanne, Switzerland; 3 Service of Dermatology, Lausanne University Hospital (CHUV), Lausanne, Switzerland

**Keywords:** COVID toes, Chilblains, Pernio, IFN type I

## Abstract

**Background:**

Since the beginning of the coronavirus disease 2019 pandemic, the most common skin lesions observed due to infection with the severe acute respiratory syndrome coronavirus 2 are pseudochilblains (or coronavirus disease toes). However, this pathology remains infrequent and difficult to diagnose, as no specific test exists.

**Case presentation:**

Two Caucasian women, 30 and 22 years old, presented to our General Medicine Unit with perniosis lesions on the feet during the first two waves of the coronavirus disease 2019 pandemic. They did not have respiratory or general symptoms of severe acute respiratory syndrome coronavirus 2 infection, the reverse transcription polymerase chain reaction on nasopharyngeal swabs was negative, and the serology was positive only in the first case. The clinical presentation differed for the two cases, as the second patient suffered from swelling and burning after cold application. The diagnosis was based on clinical presentation, temporality, exclusion of other differential diagnoses, and blood test results (positive serology in the first case and high level of CXCL13 and VEGF in the second), supported by current literature. Lesions resolved spontaneously in the first patient. The second case was hospitalized for pain management and received corticosteroid therapy with resolution of the symptoms.

**Conclusion:**

These two cases with different clinical presentations illustrate the diagnostic approach to coronavirus disease 2019, a challenging disease with diverse manifestations, including, in some cases, coronavirus disease toes. We present a literature review that illustrates the progression of scientific research. Skin lesions associated with coronavirus disease 2019 infection could be the expression of an important interferon type 1 response and should be considered in the differential diagnosis in a primary care setting.

## Background

In December 2019, a new virus was identified in Wuhan, China, called severe acute respiratory syndrome coronavirus (SARS-CoV2-). The virus spread rapidly worldwide, and the World Health Organization (WHO) declared this outbreak a pandemic on 12 March 2020 [1]. At the onset of the pandemic, COVID-19 infection was described as pneumonia that could induce respiratory failure. Epidemiological studies showed that the most frequent symptoms were fever, malaise, fatigue, cough, sputum, and dyspnea [2]. Other common symptoms included neurological symptoms, myalgia, diarrhea, headache, rhinitis, and chest pain [3]. In addition, an abnormally high number of skin lesions associated with COVID-19 were described [4].

Pseudochilblains, also called pernio or COVID toes, are the most common skin manifestations of COVID-19 disease [5]. They are mainly described in Europe and the USA but are rarely reported in Asia [6]. Depending on the studies, the prevalence of pseudochilblains in suspected or confirmed patients with COVID-19 ranges from 3.7% to 29% [4, 7]. The prevalence of any skin lesion in suspected or confirmed COVID-19 patients was 60%, of which 48% were pseudochilblains [4]. In a study with patients with confirmed COVID-19 suffering from skin lesions, the prevalence of pseudochilblains was 30% [8].

Lesions appear as acral inflammatory erythematous–violaceous macules or papules that can sometimes become blisters or swelling [9]. Clinical presentation and pathologic features are similar to idiopathic and autoimmune-related chilblains, without the same epidemiologic characteristics (no cold exposure or autoimmune disorder) and are thus called “pseudochilblains” [10]. Patients frequently show no or few symptoms of COVID-19 before or during pseudochilblains lesions, and biological tests are often negative, making it difficult to prove a link between the skin lesions and the infection. As scientific progress is being made, literature shows evidence of this link [11].

In this article, we present two cases of COVID toes, which we observed in our emergency department in Lausanne, Switzerland, during two different local waves of COVID-19.

## Case presentation

### First case

A 30-year-old Caucasian woman presented at the beginning of the SARS-CoV-2 pandemic with foot lesions. She complained of erythematous swelling and a burning sensation on the left foot that had started at *T* = 0, initially limited to the third and fourth toes and then progressed to other toes and increased in intensity. At *T* = 10 days, she developed blisters, which evolved into desquamation with a diminution of the erythema. The patient complained of tiredness but had no fever or other systemic symptoms during this period. She had no relevant past medical history or family disease.

During the clinical evaluation at *T* = 1 month, we noticed a desquamation of several toes without pain, swelling, or redness. Complete blood count and C-reactive protein were normal. A reverse transcription polymerase chain reaction (RT–PCR) test for SARS-CoV-2 on a nasopharyngeal swab was not performed due to the delay since the onset of symptoms and the absence of respiratory manifestations. The SARS-CoV-2 serology at *T* = 1 month showed a weak positive IgM level at 2.58 g/L (normal value < 2.41 g/L). A biopsy was not performed, as no skin lesions were found on examination. We retained the pseudochilblain diagnosis based on current literature supporting clinical diagnosis during the COVID-19 outbreak and after excluding other differential diagnoses [12]. Symptoms resolved spontaneously without treatment within *T* = 2 months.

### Second case

**Fig. 1:**
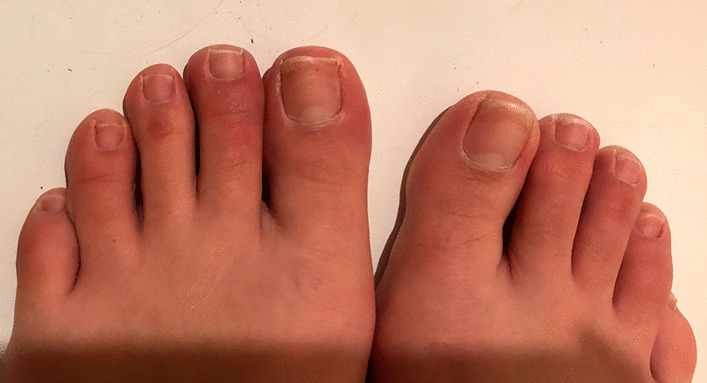
Initial clinical presentation of chilblain-like lesions (*T* = 0). Diffuse redness macules and slight swelling of the toes as initial clinical presentation

**Fig. 2:**
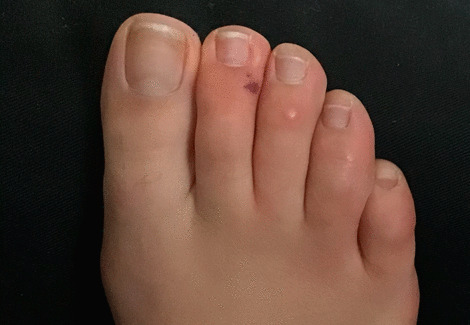
Evolution two days after the onset of symptoms (*T* = 2 days). A purpuric lesion appeared on the second right toe. Improvement of diffuse redness and edema

A 22-year-old Caucasian woman with no other comorbidities ans not vaccinated against COVID-19 presented in the second year of the SARS-CoV-2 pandemic with acute foot lesions without fever or other systemic manifestations. She reported redness, heat, swelling, and itching of all toes associated with pain at *T* = 0 (Fig. 1). The symptoms were constant, relieved by cold and worsened by heat. She described the appearance of a nodule on the right second toe that lasted for a few days (Fig. 2). Dark red punctiform lesions appeared on some toes. During the examination at *T* = 5 days, she declared that she was not relieved by topical and oral anti-inflammatory drugs, paracetamol, or antihistamine medication.

**Fig. 3:**
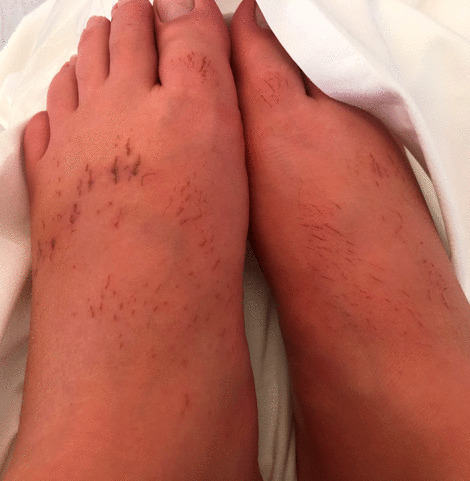
Evolution 1 month after the onset of symptoms (*T* = 1 month). Inflammatory swelling of the two feet with dark red lesions, punctiform or linear, on the back of the two feet

The SARS-CoV-2 RT–PCR on nasopharyngeal swabs at *T* = 5 and *T* = 45 days were negative. At *T* = 3 weeks, the toe edema temporarily subsided. A few days later, inflammatory swelling of the two feet reappeared with a burning sensation and diffuse dysesthesia. Other dark red lesions, punctiform or linear, appeared on the back of the two feet (Fig. 3), which became crusted.

**Fig. 4:**
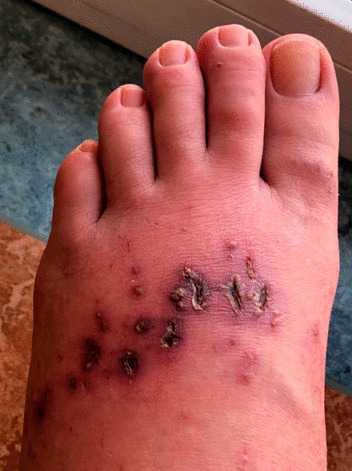
Evolution after 6 weeks after the onset of symptoms (*T* = 6 weeks). Erythematous edema with dark red, punctiform, or linear crusted lesions on the back of the feet

The patient was finally hospitalized for better pain management at *T* = 6 weeks. Clinical examination revealed warm, erythematous edema of both feet and ankles, spreading upwards over both legs’ lower third. There were dark red, punctiform, or linear lesions on the toes and back of the feet. A cyanotic border surrounded some lesions; others were cracked and had a crusty or fibrinous background (Fig. 4). The rest of the examination showed a slight decrease in pallesthesia (7.5/8), probably related to edema. A screening for autoimmune diseases and a SARS-CoV-2 serology were negative. The CXCL13 chemokine was elevated at 1021 pg/ml (normal value < 114 pg/ml), and VEGF-A was slightly increased at 663 pg/ml (normal value < 569 pg/ml). Magnetic resonance imaging (MRI) of the feet and ankles showed tenosynovitis of the long extensor digitorum tendons of the right foot, possibly secondary to adjacent tissue compression and fatty infiltration of the dorsum of both feet. Following a dermatological assessment, a skin biopsy was not performed due to extensive swelling.

Although the SARS-CoV-2 RT–PCR and serologies were negative, we retained the pseudochilblain diagnosis after excluding other differential diagnoses and considering COVID toes as a clinical diagnosis during a COVID-19 outbreak following the current literature [12]. The patient's skin lesions resembled those described in literature in association with waves of COVID-19, and we found an increase of CXCL13 chemokine in the blood, which was related to severe COVID-19 infection [13]. The diagnosis of erythromelalgia was considered unlikely due to the nonparoxysmal nature of the symptoms.

**Fig. 5:**
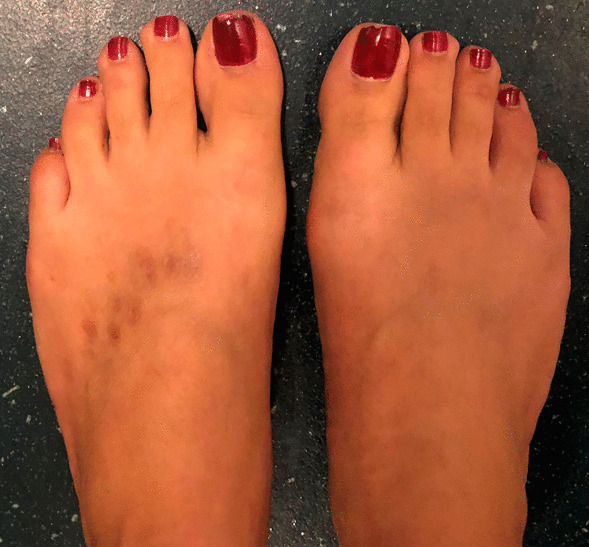
Evolution 1 year after the onset of symptoms (*T* = 1 year). No redness or swelling. There are still residual marks from the crusts on the back of the left foot

Symptoms improved significantly after a few days of oral prednisone therapy, 40 mg per day, followed by 20 mg per day for 7 days and 10 mg per day for 7 days. The patient was also treated with pregabalin and tramadol for pain relief and 300 mg of aspirin per day. The pain resolved in a few weeks, she stopped taking painkillers, and the lesions did not recur at *T* = 1 year (Fig. 5).

## Discussion

These two cases of COVID toes had different clinical presentations and evolution, but they both developed symptoms during pandemic waves within a context of high COVID-19 infection incidence, indicating a temporal link. We retained the diagnosis of pseudochilblains or COVID toes based mainly on clinical elements after excluding other differential diagnoses and following current literature for indirect evidence of this association during the COVID-19 outbreak.

The first patient presented typical symptoms and recovered within 1 month. Serology revealed positive IgM for SARS-CoV-2 without positive IgG at *T* = 1 month. Studies have shown that antibody titers appear related to disease severity, and a low or undetectable IgG rate might be related to a strong viral clearance [14].

The second patient had different interesting features of COVID toes, with swelling and a burning sensation after cold application. Although the patient had a negative serology, current literature has shown that an excessive interferon-alpha (IFN-α) response might clear the virus before humoral immunity occurs and explain the negative serology [15]. Furthermore, the second patient had a high level of CXCL13, which has been shown to be a marker of COVID-19 severity [16], and a high level of VEGF-A, an endothelial marker, that has been found in late pseudochilblains 20 days after symptom onset [17]. The evolution was slow over about 2 months and required corticosteroid therapy.

COVID toes occur mainly in children and young adults, last on average 14 days (up to 1 month) and resolve spontaneously. Despite the frequent absence or low-grade typical COVID-19 symptoms, recent contact with COVID-19 patients is often found. The skin lesions occur 2 to 4 weeks after the initial manifestation [18]. These lesions are primarily associated with mild COVID-19 with a good prognosis [19]. Laboratory tests are often negative. A study found 7% positive tests without distinguishing RT–PCR or serologies [19]. In another study, 30% of patients had positive serologies without positive RT–PCR [15]. Finally, some authors showed negative IgG with positive IgA in children in 31.6% of patients [20]. The prevalence of these lesions is not known with certainty, but it has been estimated to be between 3.7% and 29% of patients with suspected or confirmed COVID-19 [4, 7].

The formal causal link between pseudochilblains and COVID-19 remains challenging to prove. The characterization of this syndrome relies primarily on retrospective studies with many limitations, and the RT–PCR or serologies are often negative [20]. The RT–PCR is negative in most patients, probably because pseudochilblains appear late during the infection. It has also been shown that patients with COVID-19 with mild or no symptoms have lower viral loads that are more challenging to detect [15]. Also, we currently know that specific early humoral response to SARS-CoV-2 is dominated by IgA, which has a crucial role in virus neutralization. Serologies might be negative because IgA is not analyzed in routine serology [21, 22]. At last, as mentioned above, this could be related to a strong IFN-α response that clears the virus before the humoral response [15].

Type 1 IFN is known as essential for host defense against viruses and a high concentration of type 1 interferon-inducible myxovirus resistance protein A has been found in pseudochilblain biopsies, similar to the transcriptional signature of lupus [23]. Therefore, some people would have a strong type 1 IFN response that would favor rapid elimination of the virus without suffering from the disease, thus blocking seroconversion. Pseudochilblains would, therefore, be a viral-induced type I interferonopathy [24]. A study has identified a stimulator of interferon genes (STING)-dependent type I IFN signature that is primarily mediated by macrophages adjacent to areas of endothelial cell damage [25].

A recent review proposes that the mechanism of COVID toes is related to an interaction between SARS-CoV-2 cell infection via ACE2, the RAAS (renin–angiotensin–aldosterone system), sex hormones, and the IFN type I immune response [26]. In a small case series, viral particles were found in endothelial cells, suggesting that the vessel damage could be directly related to SARS-CoV2 [27]. Finally, one study compared idiopathic chilblains to pseudochilblains, demonstrating histological similarities. However, pseudochilblains showed a systemic response with a high prevalence of ANCA, IgA, and IFN type 1 and an endothelial alteration [17].

## Conclusion

Pseudochilblain or COVID toes is a recent disease that emerged with the COVID-19 pandemic. Current literature suggests pathophysiological mechanisms related to a cutaneous expression of an important IFN type 1 response. The typical clinical presentation, history, and examination, the consideration and exclusion of other differential diagnoses, the absence of a history of perniosis, and the timing of COVID waves allow the diagnosis of pseudochilblains following a COVID-19 infection. Clinicians in primary care should be aware of chilblain-like lesions and suspect COVID-19 infection, even in the presence of a negative RT–PCR test and negative serology.

## Data Availability

All data generated or analyzed during this study are included in this published article.
